# The Simultaneous Model-Based Estimation of Joint, Muscle, and Tendon Stiffness is Highly Sensitive to the Tendon Force-Strain Relationship

**DOI:** 10.1109/TBME.2023.3324485

**Published:** 2024-02-26

**Authors:** Christopher P. Cop, Kristen L. Jakubowski, Alfred C. Schouten, Bart Koopman, Eric J. Perreault, Massimo Sartori

**Affiliations:** Department of Biomechanical Engineering, University of Twente 7522 LW Enschede, The Netherlands; Department of Biomedical Engineering, Emory University, USA, and also with the Georgia Institute of Technology, USA.; Department of Biomechanical Engineering, Delft University of Technology, The Netherlands.; Department of Biomechanical Engineering, University of Twente, The Netherlands.; Department of Biomedical Engineering, Northwestern University, USA; Shirley Ryan Ability Lab, USA, and also with the Department of Physical Medicine and Rehabilitation Northwestern University, USA.; Department of Biomechanical Engineering, University of Twente, The Netherlands.

**Keywords:** Joint stiffness, muscle stiffness, musculoskeletal modeling, tendon stiffness

## Abstract

**Objective:**

Accurate estimation of stiffness across anatomical levels (i.e., joint, muscle, and tendon) in vivo has long been a challenge in biomechanics. Recent advances in electromyography (EMG)-driven musculoskeletal modeling have allowed the non-invasive estimation of stiffness during dynamic joint rotations. Nevertheless, validation has been limited to the joint level due to a lack of simultaneous in vivo experimental measurements of muscle and tendon stiffness.

**Methods:**

With a focus on the triceps surae, we employed a novel perturbation-based experimental technique informed by dynamometry and ultrasonography to derive reference stiffness at the joint, muscle, and tendon levels simultaneously. Here, we propose a new EMG-driven model-based approach that does not require external joint perturbation, nor ultrasonography, to estimate multi-level stiffness. We present a novel set of closed-form equations that enables the person-specific tuning of musculoskeletal parameters dictating biological stiffness, including passive force-length relationships in modeled muscles and tendons.

**Results:**

Calibrated EMG-driven musculoskeletal models estimated the reference data with average normalized root-mean-square error ≈ 20%. Moreover, only when calibrated tendons were approximately four times more compliant than typically modeled, our approach could estimate multi-level reference stiffness.

**Conclusion:**

EMG-driven musculoskeletal models can be calibrated on a larger set of reference data to provide more realistic values for the biomechanical variables across multiple anatomical levels. Moreover, the tendon models that are typically used in musculoskeletal modeling are too stiff.

**Significance:**

Calibrated musculoskeletal models informed by experimental measurements give access to an augmented range of biomechanical variables that might not be easily measured with sensors alone.

## I. Introduction

MOVEMENT disorders such as those arising from stroke [1], cerebral palsy [2], spinal cord injury [3], or chronic pain [4] dramatically disrupt musculoskeletal impedance at all anatomical levels, i.e., joint, muscle and tendon. Impedance is the dynamic relationship between an imposed displacement and the resultant force or torque [5]. Neurorehabilitation should aim at re-establishing normative musculoskeletal impedance- and force-generating capacity for an individual person [6]. For instance, clinicians might need to understand what muscles actively and passively contribute to a joint’s reduced range of motion [7] to inform personalized surgical, robotic or physical training interventions. Consequently, a fundamental challenge in the fields of biomechanics, motor control, and physical rehabilitation has long been measuring muscle impedance noninvasively, using minimal experimental setups, across a broad range of functionally relevant conditions in health and disease [8].

Experiment-based approaches for the estimation of joint impedance have been proposed for controlled movements in a laboratory setup [9], [10]. They combine measurements from sensorized robotic manipulators, that are used to apply external perturbations to an individual’s biological joint, with system identification algorithms to estimate joint-level biomechanical variables in an accurate way. Recent work incorporated muscle ultrasonography and electromyography (EMG) measurements, leading to a novel methodology to identify the contributions of muscle and tendon to the net joint impedance and stiffness, or position-dependent component of impedance [11], via robotic-induced perturbations to the target biological joint. However, direct measurements are time-consuming and require a complex laboratory setup. Additionally, the need for external joint perturbations and specialized equipment, such as dynamometers and ultrasonography, limits measurements to highly controlled motor tasks that are often not representative of daily movements and decreases translation to clinical or day-to-day settings where complex instrumentation may not be viable.

EMG-driven modeling is a computational tool that has been widely used to estimate muscle-tendon unit (MTU) forces and resulting joint torques from experimentally recorded EMGs and joint angles [12], [13]. In this context, it is critical to derive closed-form equations that capture relevant MTU passive and contractile dynamics, as well as to calibrate underlying model parameters to best estimate experimentally recorded joint torques [12], [14]. Our recent developments in EMG-driven musculoskeletal modeling enabled, for the first time, the simultaneous estimation of joint torque and stiffness during dynamic joint rotations in intact humans in vivo [15]. Importantly, the proposed framework allowed the estimation of joint stiffness without the need of external perturbations, which enabled, for the first time, the study of biological stiffness both in presence and absence of external perturbations. Consequently, the effect that external perturbations have on joint stiffness could be assessed.

However, it is unclear to what extent an EMG-driven model that has been fitted to joint-level biomechanical variables can estimate muscle- and tendon-level variables, such as muscle and tendon stiffness and muscle displacement. Previous validations of muscle and tendon properties estimated from EMG-driven models of human movement have been limited by the lack of reference muscle- and tendon-level biomechanical variables. Joint-level accuracy does not necessarily ensure muscle-level accuracy, as muscle-level phenomena, such as short-range stiffness, might not be reflected at the joint level because each joint is spanned by multiple agonist and antagonist muscles, i.e., muscle redundancy [16]. A new set of closed-form equations and the calibration of additional MTU parameters might be required to capture muscle dynamics.

In this study, we propose an EMG-driven musculoskeletal model that relies on a set of closed-form equations that enables the person-specific tuning of musculoskeletal features that influence stiffness at multiple anatomical levels, i.e., joint, muscle, and tendon levels. This enables adjustments in tendon stiffness and muscle passive stiffness, in addition to other force-generating parameters, i.e., muscle optimal fiber length, tendon slack length, maximum isometric force, pennation angle at optimal fiber length, and a “shape factor” to non-linearly scale measured EMGs to obtain muscle activations. With a focus on the triceps surae, we systematically validate our proposed approach at the joint, muscle, and tendon levels against reference stiffness data derived via system identification informed by perturbation-based dynamometry and ultrasonography. We demonstrate that tendon units widely modeled in the literature employed too stiff force-strain characteristics and that modeling a more compliant tendon is critical for the estimations of stiffness across anatomical levels in EMG-driven models.

Our proposed methodology enables multi-level stiffness estimation across a wide repertoire of movements. Moreover, it does not require joint perturbations, nor ultrasonography, to estimate stiffness across anatomical levels, thus facilitating the translation of this technology to the clinics, e.g., to guide rehabilitation interventions, and out of the lab.

## II. Methods

### A. Participants

Twelve healthy volunteers (age range: 26–36 years, 6 males) with no self-reported history of neurological or ankle impairments participated in this study. All participants tested right leg dominant using the Waterloo Footedness questionnaire. The Northwestern University Institutional Review Board approved the experimental procedures (STU00009204 and STU00213839) and all subjects provided written informed consent. The experiments complied with the Declaration of Helsinki.

### B. Apparatus

**Fig. 1.**
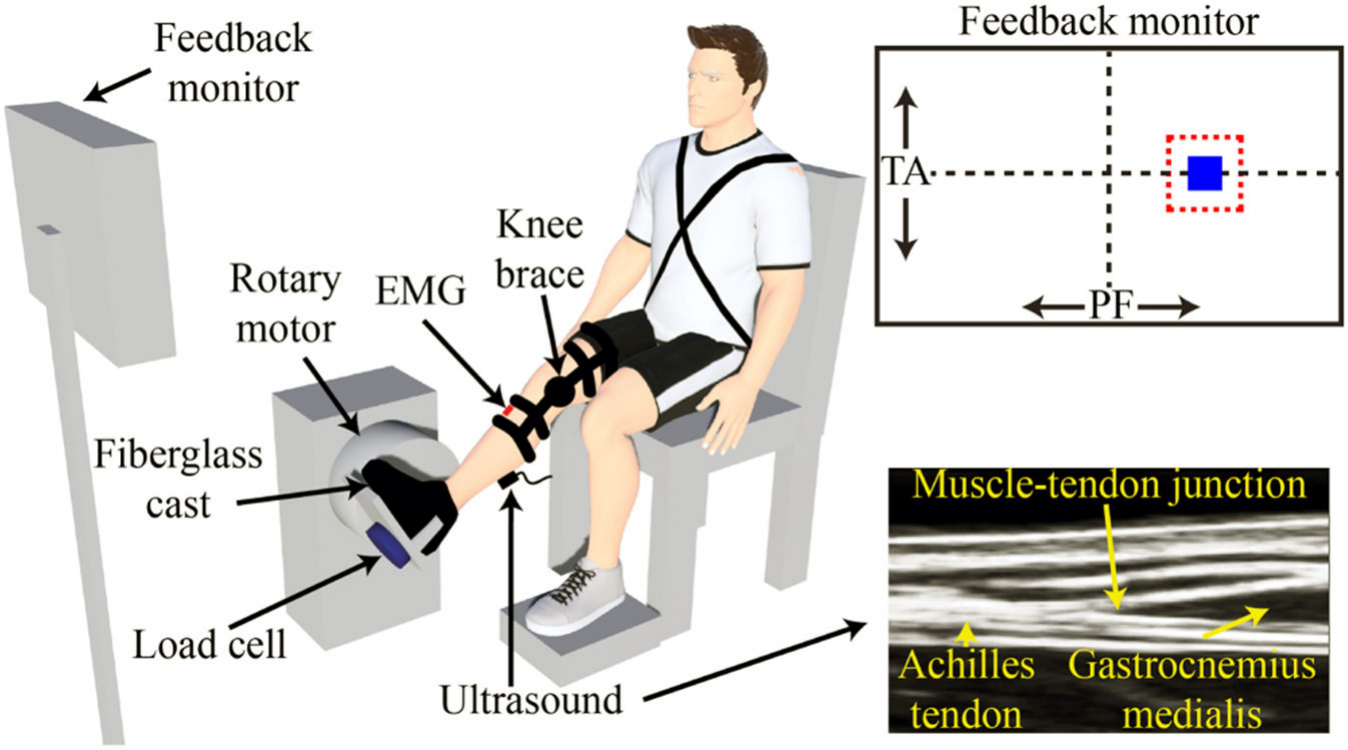
Schematic of the experimental setup. The participant’s foot was secured to the rotary motor via a custom-made cast. Ankle angle was rigidly controlled by the rotary motor while a 6-degree-of-freedom load cell measured the resultant ankle torque. B-mode ultrasound was used to image the muscle-tendon junction of the medial gastrocnemius. A knee brace prevented any unwanted knee flexion or extension. Participants were provided real-time visual feedback of their mean plantarflexor (PF) and tibialis anterior (TA) EMG. Figure adapted from Jakubowski et al. 2023 [17].

Fig. 1 summarizes the experimental setup. Participants were seated in an adjustable chair (Biodex Medical Systems, Inc. Shirley, NY, USA) with their right leg extended in front of them. The knee was stabilized at 15° of flexion with a brace (Innovator DLX, Ossur, Reykjavik, Iceland), which prevented movement at the proximal end of the biarticular gastrocnemius medialis (GM) and gastrocnemius lateralis (GL). We rigidly secured the participant’s foot to an electric rotary motor (BSM90N-3150AF, Baldor, Fort Smith, AR, USA) via a custom-made fiberglass cast. The cast completely encased the foot while preserving the full range-of-motion of the ankle. We aligned the ankle center of rotation in the sagittal plane with the center of rotation of the motor and restricted all movement and rotation to the sagittal plane. Electrical and mechanical safety stops limited the rotation of the motor within the participant’s range of motion. An encoder (24-bit, PCI-QUAD04, Measurement Computing, Norton, MA). integrated within the motor measured ankle angle, while a six-degree-of-freedom load cell (45E15A4, JR3, Woodland, CA, USA) measured all ankle forces and torques. xPC Target (MATLAB, Mathworks, Natick, MA) controlled the motor in real-time. We used a position control scheme such that the motor dictated the position of the participant’s ankle at all times.

EMG data were collected at 2500 Hz from the GM, GL, soleus (SO), and tibialis anterior (TA) using single differential bipolar surface electrodes (Bagnoli, Delsys Inc, Boston, MA, 10 mm interelectrode distance). Standard skin preparation techniques were used before applying each electrode to the skin [18]. Electrodes were placed on the belly of the respective muscle. All kinematic, kinetic, and EMG data were passed through an antialiasing filter (500 Hz using a 5-pole Bessel filter) and sampled at 2.5 kHz using a 24-bit data acquisition system (PCI-6289, Measurement Computing, Norton, MA, USA). EMG data were collected for the visual feedback provided to the subjects and to drive the musculoskeletal model.

We rigidly secured a B-mode ultrasound probe (LV7.5/60/128Z-2 Telemed, Lithuania) to the leg to image the GM muscle-tendon junction (MTJ). We have demonstrated previously that the results during active isometric contractions do not vary when imaging the various triceps surae muscles (GM vs. GL vs. SO) [11]. We positioned the probe parallel to the muscle belly (longitudinally) such that the MTJ was centered on the image. Ultrasound data had a mean frame rate of 124 Hz and were synchronized with all measurements from the rotary motor.

### C. Experimental Data

#### 1) Protocol:

Before starting the experiment, participants performed maximum voluntary contractions (MVC) trials to obtain EMG normalization factors. Participants completed three MVC trials in both plantarflexion and dorsiflexion directions with the ankle angle fixed at 10° of plantarflexion, each lasting 10 s. Our primary objective was to quantify ankle, muscle, and tendon stiffness during movement. Therefore, the participant’s ankle was moved through a sinusoidal motion with an amplitude of 20° and a frequency of 0.5 Hz. The movement was centered at 10° of plantarflexion. Small rotational perturbations were superimposed on the large sinusoidal movement. We used pseudo-random binary sequence (PRBS) perturbations with an amplitude of 0.14 rad, a maximum velocity of 1.75 rad/s, and a switching time of 153 ms. Twenty-one trials were collected, each lasting 40 s. This large number was needed for the time-varying system identification described below. During each trial, participants were instructed to produce and sustain plantarflexor EMG activity at 20% of MVC. The plantarflexor EMG activity was defined as the normalized mean of the EMG from the GM, GL, and SO, the major ankle plantarflexors. Real-time visual feedback of plantarflexor EMG was shown on a screen. We also provided TA EMG feedback to prevent co-contraction (Fig. 1). Practice was allowed so participants could become proficient with the task. Rest breaks were provided between each trial to prevent fatigue.

#### 2) Signal Processing:

All data processing and analysis was completed using custom-written software in MATLAB. The same experimenter manually digitized the MTJ within each frame of the ultrasound video. Ultrasound data were synchronized with all other data [19], and linearly interpolated to the sampling rate of all other data (2.5 kHz).

To obtain normalized EMG envelopes, raw EMG recordings were band-pass filtered with a fourth order Butterworth notch filter (cutoff frequencies: [59 61] Hz) to remove the 60 Hz powerline interference, demeaned, rectified, smoothed using a moving mean window of 250 ms, and normalized by the maximum value of the MVC recording.

Prior to further processing, all data were decimated to 100 Hz.

#### 3) Ankle, Muscle, and Tendon Stiffness:

**Fig. 2.**
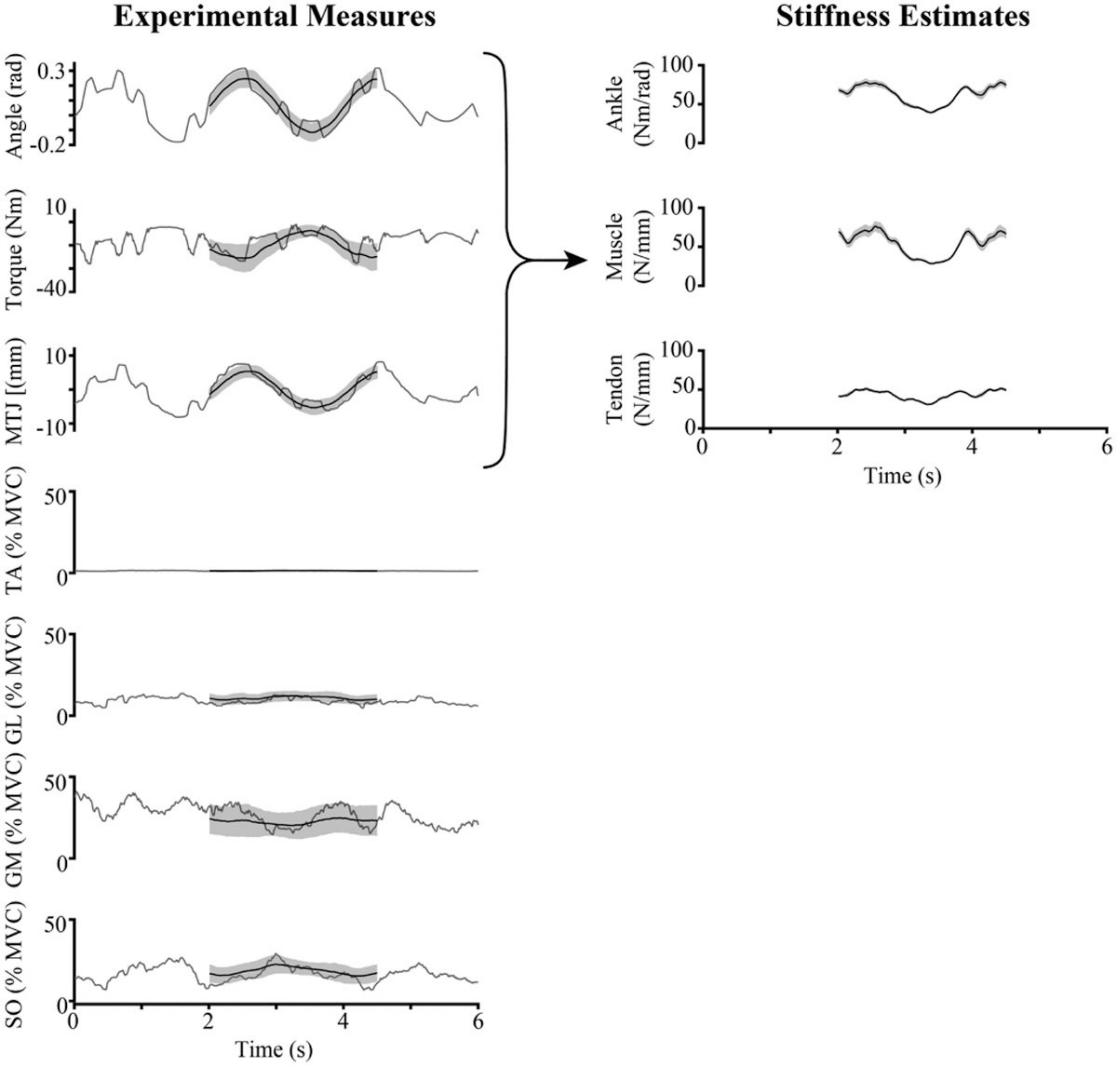
Sample data from a representative subject. There are 6-second snippets of ankle angle, torque, gastrocnemius medialis (GM) muscle-tendon junction (MTJ) displacement, and electromyography (EMG) data, from a single realization (gray) and the mean from the 200 realizations (black). The shaded black region is the standard deviation across the 200 realizations. These data were used to obtain a task-specific estimation of ankle, muscle, and tendon stiffness.

Ankle, muscle, and tendon impedance and stiffness were estimated from the experimentally measured ankle angle, ankle torque, and MTJ displacement (Section II-B) via non-parametric system identification (Fig. 2).

To calculate impedance during time-varying conditions, the system identification algorithm requires multiple repetitions of repeated data [20]. Therefore, all data were segmented into overlapping three-period long segments. Each segment started one period after the previous one. The realization was removed if the TA was active. The TA was deemed active if the activation within the realization exceeded 5% MVC. We used the 200 realizations where the mean plantarflexor EMG had the lowest mean-squared error relative to 20% MVC (the targeted activation).

To quantify ankle, muscle, and tendon impedance, we used our recently developed method [11]. Briefly, the experimental measures of ankle angle, ankle torque, and MTJ displacement were used in these calculations. Ankle impedance was quantified from the relationship between the imposed ankle rotations and the resultant ankle torque [5]. We assumed that the muscle and tendon are connected in series [21], and muscle-tendon unit displacement can be determined by the rotation of the ankle multiplied by the Achilles tendon moment arm. Moreover, we assume that the proximal end of the muscle is fixed, and, thus, any movement of the MTJ is a measure of muscle length change. Based on these assumptions, muscle and tendon impedance can be estimated from the estimates of ankle impedance and the translation ratio–the relationship between MTJ displacement and the angular rotations of the ankle. Specifically, to estimate ankle impedance and the translation ratio, we used a non-parametric time-varying system identification algorithm [20]. The algorithm computed the time-varying impulse response functions (IRFs) for ankle impedance and the translation ratio at each time point along the movement profile. The stiffness, or position-dependent component of ankle impedance, and the translation ratio were computed by integrating the IRFs. From the estimates of ankle stiffness and the static translation ratio, we estimated muscle and tendon stiffness algebraically [11].

We used a bootstrapping procedure to calculate the confidence intervals for our ankle, muscle, and tendon stiffness estimates. The 200 realizations were sampled randomly with replacement to produce a new ensemble of 200 realizations. The new ensemble was then used to compute stiffness. We repeated this procedure 100 times, resulting in a distribution of stiffnesses.

A single approximation of the Achilles tendon moment arm of 51.3 mm was used for all analyses. The moment arm was estimated as the mean across subjects from Clarke et al. [22] with an ankle angle of 10° of plantarflexion.

### D. EMG-Driven Musculoskeletal Model

**Fig. 3.**
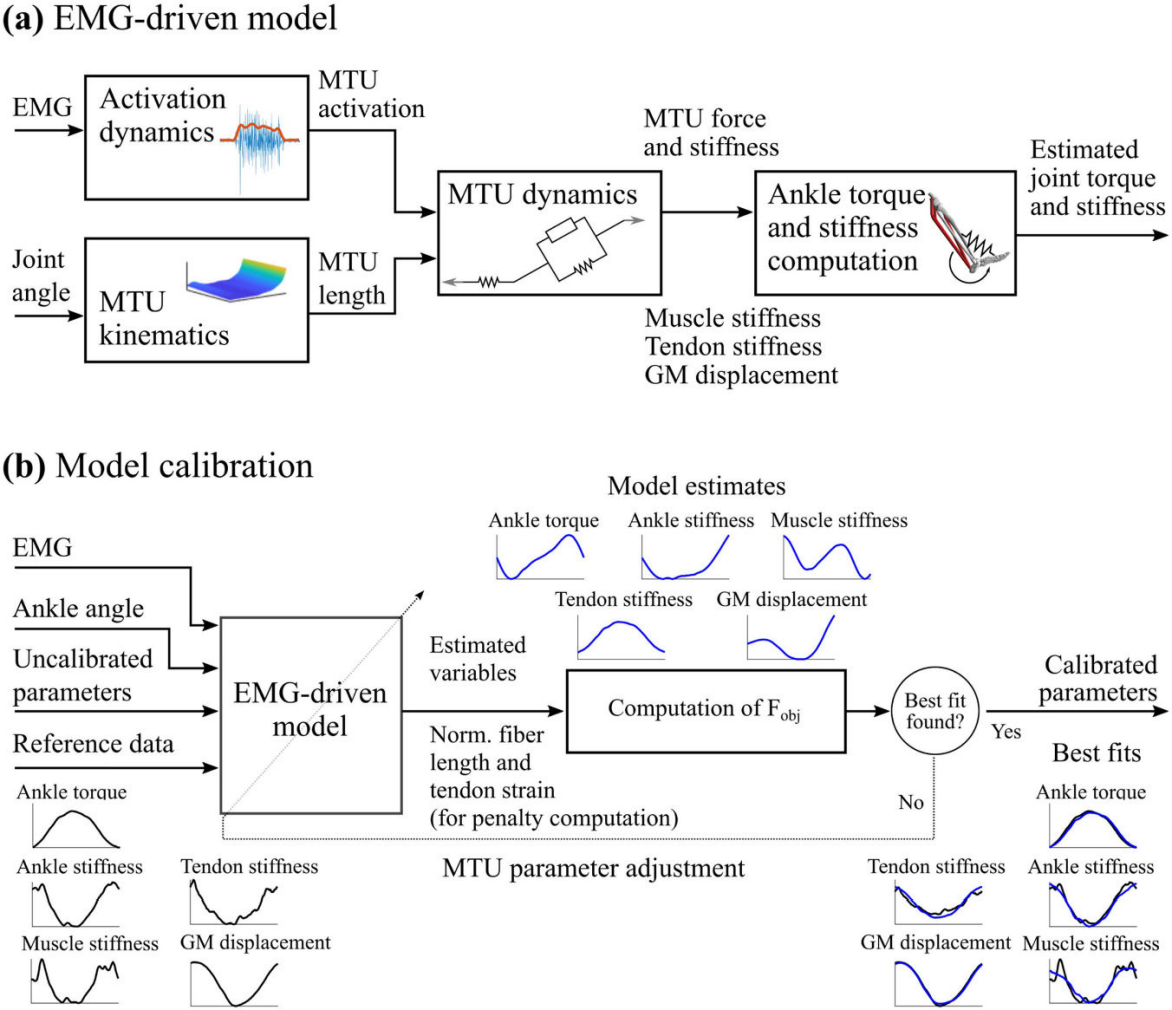
(a) EMG-driven model: the “Activation dynamics” block maps experimental muscle excitations into muscle-tendon unit (MTU) activations. The “MTU kinematics” block maps ankle plantar-dorsiflexion angle into MTU length. The “MTU dynamics” block estimates MTU force and stiffness employing a Hill-type muscle model driven by MTU activation and length with an elastic tendon. The “Ankle torque and stiffness computation” block projects MTU force and stiffness onto the the joint level via the MTU moment arm to obtain estimates of joint torque and stiffness. (b) Model calibration: Seven parameters per MTU, namely optimal fiber length, tendon slack length, maximum isometric force, shape factor, pennation angle at optimal fiber length, stiffness of the tendon force-strain curve and stiffness of the muscle passive force-length curve, are adjusted to best track input reference ankle torque, ankle stiffness, GM displacement, muscle stiffness, and tendon stiffness profiles using the EMG-driven model described in (a). A simulated annealing optimization routine is used to adjust MTU parameters to minimize the difference between reference (plots in black) and estimated (plots in blue) biomechanical variables.

This work extends the EMG-driven modeling framework we recently developed [15]. We introduce an extended set of closed-form equations to estimate forces and stiffness across multiple anatomical levels, i.e., joint, tendon, and muscle levels. This new formulation and the reference data set at multiple anatomical levels enable, for the first time, the calibration of the stiffness of the modeled tendons and the muscle passive stiffness. To best match the assumptions of the experimental approach II-C3, the EMG-driven model used in this study comprises three MTUs with elastic tendons: GM, GL, and SO. The EMG-driven modeling pipeline (Fig. 3) is outlined below.

#### 1) Activation Dynamics:

Muscle excitations, u, here defined as the normalized EMG envelopes, are mapped into MTU activations (a) without an intermediate muscle fiber twitch model using the following equation:

(1) $$a=\frac{e^{Au}-1}{e^{A}-1}$$

where A∈(-3,0) is a MTU-specific parameter named shape factor that scales the level of muscle co-contraction.

#### 2) MTU Kinematics:

Joint angles are mapped into MTU length using a set of multi-dimensional B-splines [23]. To be consistent with the assumptions made in the experimental approach [11], a constant moment arm, r, (r=51.3mm [22]) was used for all modeled muscles.

#### 3) MTU Dynamics:

MTU force, $F^{MTU}$, is computed using a Wijngaarden–Dekker–Brent optimization to solve the equilibrium equation between tendon force, $F^{T}$, and muscle fiber force, $F^{M}$:

(2) $$F^{MTU}=F^{T}=F^{M}\;\cos\;\phi$$

where ϕ is the MTU’s pennation angle, that is computed using the following expression, assuming a constant muscle thickness:

(3) $$\phi =\arcsin\left(\frac{\sin\;\phi_{o}}{{\tilde{l}}^{M}}\right)$$

where ${\tilde{l}}^{M}$ is normalized muscle fiber length (${\tilde{l}}^{M}=l^{M}∕l_{o}^{M}$, with $l^{M}$ and $l_{o}^{M}$ being muscle fiber length and muscle optimal fiber length, respectively) and $\phi_{o}$ is the MTU’s pennation angle at $l_{o}^{M}$.

$F^{T}$ is computed using a generic dimensionless tendon force strain relationship, $f_{t}(\epsilon^{T})$ (adapted from [24]), where $\epsilon^{T}$ is tendon strain ($\epsilon^{T}=l^{T}∕l_{s}^{T}\;\text{-}\;1$, with $l^{T}$ and $l_{s}^{T}$ being tendon length and tendon slack length, respectively), scaled by the MTU’s maximum isometric force, $F_{max}$:

(4) $$F^{T}=F_{max}f_{t}(\epsilon^{T})$$

(5) $$f_{t}(\epsilon^{T})=G_{t}\;(a_{1}\;\text{exp}\;[a_{2}\;(\epsilon^{T}+a_{3})]-a_{4})$$

where $G_{t}\in (0.05,1.5)$ is a newly introduced MTU parameter that scales the tendon stiffness. The values of the coefficients $a_{1}$, $a_{2}$ $a_{3}$ and $a_{4}$ can be found in Table III (Appendix A).

$F_{M}$ is computed as a function of a, ${\tilde{l}}^{M}$, and normalized muscle contraction velocity, ${\tilde{v}}^{M}\;({\tilde{v}}^{M}=v^{M}∕v_{max}$, with $v^{M}$ and $v_{max}=10\;l_{o}^{M}∕s$ being muscle contraction velocity and maximum contraction velocity, respectively), using generic dimensionless active force-length, $f_{a}({\tilde{l}}^{M})$ (the sum of three gaussian functions, adapted from [24] to best match the cubic spline used in [15], [25]), force-velocity, $f_{v}({\tilde{v}}^{M})$ (adapted from [26] to best match the cubic spline used in [15], [25]), and passive force-length, $f_{p}({\tilde{l}}^{M})$, relationships, scaled by $F_{max}$:

(6) $$F^{M}=F_{max}\left(af_{a}({\tilde{l}}^{M})f_{v}({\tilde{v}}^{M})+f_{p}({\tilde{l}}^{M})\right)$$

(7) $$f_{a}({\tilde{l}}^{M})=\sum_{i=1}^{3}b_{1i}\;\text{exp}\left[\frac{-0.5{\left({\tilde{l}}^{M}-b_{2i}\right)}^{2}}{{\left(b_{3i}+b_{4i}{\tilde{l}}^{M}\right)}^{2}}\right]$$

(8) $$f_{v}({\tilde{v}}^{M})=c_{1}-\frac{c_{1}}{1+\text{exp}\left[\frac{c_{2}-{\tilde{v}}^{M}}{c_{3}}\right]}$$

(9) $$f_{p}({\tilde{l}}^{M})=G_{p}d_{1}{\left({\tilde{l}}^{M}\right)}^{11}$$

where $G_{p}\in (0.7,1.3)$ is a newly introduced MTU parameter that scales the muscle passive stiffness. The values of the coefficients $b_{1i}$, $b_{2i}\;b_{3i}$, $b_{4i}$, for i=1⋯3, $c_{1}$, $c_{2}$, $c_{3}$, and $d_{1}$ can be found in Table III (Appendix A).

MTU stiffness, $K^{MTU}$, is computed as the series arrangement of the tendon’s stiffness, $K^{T}$, and the equivalent muscle fiber’s stiffness in the tendon’s line of action, $K_{eq}^{M}$, [15]:

(10) $$K^{MTU}={\left(K^{T-1}+K_{eq}^{M^{-1}}\right)}^{-1}$$

$K^{T}$ is computed as:

(11) $$K^{T}=\frac{F_{max}}{l_{s}^{T}}k_{t}(\epsilon^{T})$$

with $k_{t}(\epsilon^{T})=\frac{{df}_{t}(\epsilon^{T})}{d\epsilon^{T}}$.

$K_{eq}^{M}$ is computed as [27]:

(12) $$K_{eq}^{M}=K^{M}\;\cos^{2}\;\phi +\frac{F^{M}}{l^{M}}\;\sin^{2}\phi$$

where

(13) $$K^{M}=\frac{F_{max}}{l_{o}^{M}}\left(ak_{a}({\tilde{l}}^{M})f_{v}({\tilde{v}}^{M})+k_{p}({\tilde{l}}^{M})\right)$$

with $k_{a}({\tilde{l}}^{M})=\frac{{df}_{a}({\tilde{l}}^{M})}{d{\tilde{l}}^{M}}$ and $k_{p}({\tilde{l}}^{M})=\frac{{df}_{p}({\tilde{l}}^{M})}{d{\tilde{l}}^{M}}$.

#### 4) Ankle Torque and Stiffness Computation:

Forces of the three modeled MTUs are projected via r into the joint level to obtain ankle torque $\tau^{A}$:

(14) $$\tau^{A}=\sum_{j=1}^{3}rF_{j}^{MTU}$$

where $F_{j}^{MTU}$ represents the force of the jth MTU spanning the joint.

The net ankle joint (rotational) stiffness, $K^{A}$, is computed as:

(15) $$K^{A}=\sum_{j=1}^{3}r^{2}K_{j}^{MTU}$$

where $K_{j}^{MTU}$ represents the stiffness of the jth MTU spanning the joint.

#### 5) Model Calibration Across Anatomical Levels:

For each subject, seven parameters per MTU, i.e., $l_{o}^{M}$, $l_{s}^{T}$, $F_{max}$, A, $\phi_{o}$, $G_{t}$, and $G_{p}$, are calibrated using a simulated annealing optimization routine [28] that minimizes the following multi-term objective function:

(16) Fobj=avg(T1EτA+T2EKA+T3EΔGM++T4((EKATT+EKTSM))p

where $E_{\tau^{A}}$, $E_{K^{A}}$, $E_{\Delta^{GM}}$, $E_{K_{AT}^{T}}$, and $E_{K_{TS}^{M}}$ are the mean squared errors, normalized by the variance of the reference signal, between reference and estimated ankle torque, ankle stiffness, GM displacement, Achilles tendon stiffness, and triceps surae muscle stiffness, respectively, $T_{1}$, $T_{2}$, $T_{3}$, and $T_{4}$ are weighting coefficients that determine the contribution of each biomechanical variable to the objective function, and p≥1 is a penalty factor that constrains MTUs to operate within a physiological range, i.e., p penalizes normalized muscle lengths (${\tilde{l}}^{M}<0.65$ or ${\tilde{l}}^{M}>1.35$) and negative tendon strains ($l^{T}<l_{s}^{T}$).

### E. Data Analysis

The reference data set included ankle torque, ankle stiffness, displacement of the GM MTJ, Achilles tendon stiffness, and triceps surae muscle stiffness. Our EMG-driven modeling framework explicitly computes ankle torque and ankle stiffness. As an approximation for the displacement of the GM MTJ, changes in modeled GM fiber length in the direction of the tendon’s line of action were computed ($\Delta_{GM}=l_{GM}^{M}\;\cos\;\phi_{GM}$) and centered around 0. Assuming that all modeled triceps surae muscles are in parallel, approximations for Achilles tendon stiffness, $K_{AT}^{T}$, and triceps surae muscle stiffness, $K_{TS}^{M}$, were computed by summing the tendon stiffness of the three modeled muscles ($K_{AT}^{T}=\sum_{j=1}^{3}K_{j}^{T}$) and the muscle stiffness of the three modeled muscles ($K_{TS}^{M}=\sum_{j=1}^{3}K_{j}^{M}$), respectively.

A calibration data set was created by averaging all 200 repetitions of the experiment to obtain reference ankle torque and GM displacement, and by averaging all 100 estimates of reference ankle stiffness, muscle stiffness, and tendon stiffness. A validation data set of 15 different trials was created by averaging 15 subsets of 100 randomly selected repetitions of experimental measurements of ankle torque and GM displacement, and by averaging 15 subsets of 50 randomly selected estimates of reference ankle stiffness, muscle stiffness, and tendon stiffness.

For each subject, a generic OpenSim model [29] was linearly scaled to match their height. To ensure each MTU’s operating range was preserved after linear scaling, the values for $l_{o}^{M}$ and $l_{s}^{T}$ were optimized using a previously proposed method [30]. We refer to this model as “Uncalibrated”. Lastly, each MTU’s $l_{o}^{M}$, $l_{s}^{T}$, $F_{max}$, A, $\phi_{o}$, $G_{t}$, and $G_{p}$ were further adjusted using our proposed calibration procedure (Section II-D5) to best fit reference data (Fig. 3). In this study, the weighting coefficients $T_{1}$, $T_{2}$, $T_{3}$, and $T_{4}$ were binary. In this regard, four different EMG-driven model calibration types were defined based on what reference data were used to inform the calibration. These were chosen to represent experiments of different complexity from measuring only joint torque to measuring all joint and muscle variables available in our data set. We refer to the resulting calibrated models as “Type 1”, “Type 2”, “Type 3”, and “Type 4”. Table I describes each calibration type. MTU parameters were constrained to the following ranges of values: $l_{o}^{M}$, $l_{s}^{T}$, and $\phi_{o}$ could vary 50% from their initial value, $F_{max}$ could vary from 30% to 250% of the starting value, A∈(-3,0), and $G_{p}\in (0.7,\;1.3)$. Regarding $G_{t}$, two different prior tendon force-strain curves were investigated:

1. The curve adapted from De Groote et al. [24] (Eq. 5 with $G_{t}=1$ as prior, with $G_{t}\in (0.05,\;1.5)$ during calibration).
2. A lower stiffness tendon defined as the average tendon force-strain curve across all Type 4 calibrated EMG-driven models using the aforementioned De Groote tendon as prior. The resulting tendon force-strain curve was defined by $G_{t}=0.278$, and was allowed to vary ± 30% during calibration, i.e., $G_{t}\in (0.195,0.362)$.

Each EMG-driven model was calibrated using the calibration data set. All calibrations were performed on a 64-core processor (AMD Ryzen Threadripper 3990X) and 128 GB RAM workstation, with computation times of approximately 20 minutes per calibration.

Per subject, five EMG-driven models, i.e., Uncalibrated, Type 1, Type 2, Type 3, and Type 4, were then used to estimate ankle torque, ankle stiffness, triceps surae muscle stiffness, Achilles tendon stiffness, and GM displacement using the validation data set, i.e., different EMGs and ankle angles to those employed for calibration.

Estimated biomechanical variables resulting from EMG-driven modeling simulations were compared to reference data from our experimental approach by computing the root-mean-square error normalized by the root-mean-square of the reference (nRMSE).

## III. Results

The performance of all calibration types using both tendon force-strain curves as prior (Section II-E) was assessed by comparing estimated biomechanical variables to reference values. Table II summarizes average nRMSE across all subjects for each biomechanical variable and for each EMG-driven model. The highest average nRMSE was found for the Uncalibrated model using the De Groote 2016 tendon as prior (nRMSE = 170.1 ± 63.0%), and the lowest average nRMSE was found for the Type 4 model using the De Groote 2016 tendon as prior (nRMSE = 22.7 ± 7.0%). Average nRMSE decreased with increasing calibration complexity, i.e., using more reference data to inform the calibration. For each biomechanical variable, average nRMSE was lowest when that specific biomechanical variable was first introduced in the calibration. In this regard, average ankle torque nRMSE was lowest for Type 1 model, average ankle stiffness nRMSE was lowest for Type 2 model, average GM displacement nRMSE was lowest with the Type 3 model, and average Achilles tendon stiffness and triceps surae muscle stiffness nRMSEs were lowest with the Type 4 model.

**Fig. 4.**
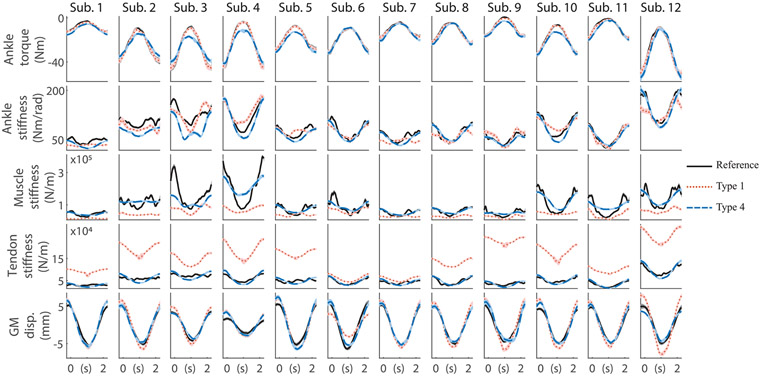
Average ankle torque (first row), ankle stiffness (second row), triceps surae muscle stiffness (third row), Achilles tendon stiffness (fourth row), and GM displacement (fifth row) time profiles for each subject. Reference values, i.e., dynamometer measurements for the ankle torque, system identification estimations obtained from perturbation-based data for joint stiffness, system identification estimations obtained from perturbation-based data in combination with ultrasound measurements for muscle and tendon stiffness, and ultrasound measurements for GM displacement, are depicted in black (solid line), estimations from the Type 1 calibrated EMG-driven model are depicted in red (dotted line), and estimations from the Type 4 calibrated EMG-driven model are depicted in blue (dashed line). Results expressed as mean values (line) ± standard deviation (shaded area). Please note that because of how the validation data set was created (Section II-E), standard deviations are in the order of magnitude of the line thickness of the mean.

Fig. 4 shows, for each subject, the average time profiles of all five biomechanical variables obtained using the EMG-driven model calibrated only on reference joint torque, i.e., Type 1, and using the best performing calibrated EMG-driven model, i.e., Type 4. On average, nRMSEs for ankle torque, ankle stiffness, triceps surae muscle stiffness, Achilles tendon stiffness, and GM displacement were 2.5 ± 1.7%, 19.3 ± 6.5%, 58.7 ± 17.7%, 197.5 ± 134.2%, and 38.9 ± 8.7%, respectively, for the Type 1 model, and 17.6 ± 5.8%, 17.8 ± 8.1%, 24.1 ± 15.0%, 16.4 ± 4.2%, and 25.5 ± 8.1%, respectively, for the Type 4 model.

**Fig. 5.**
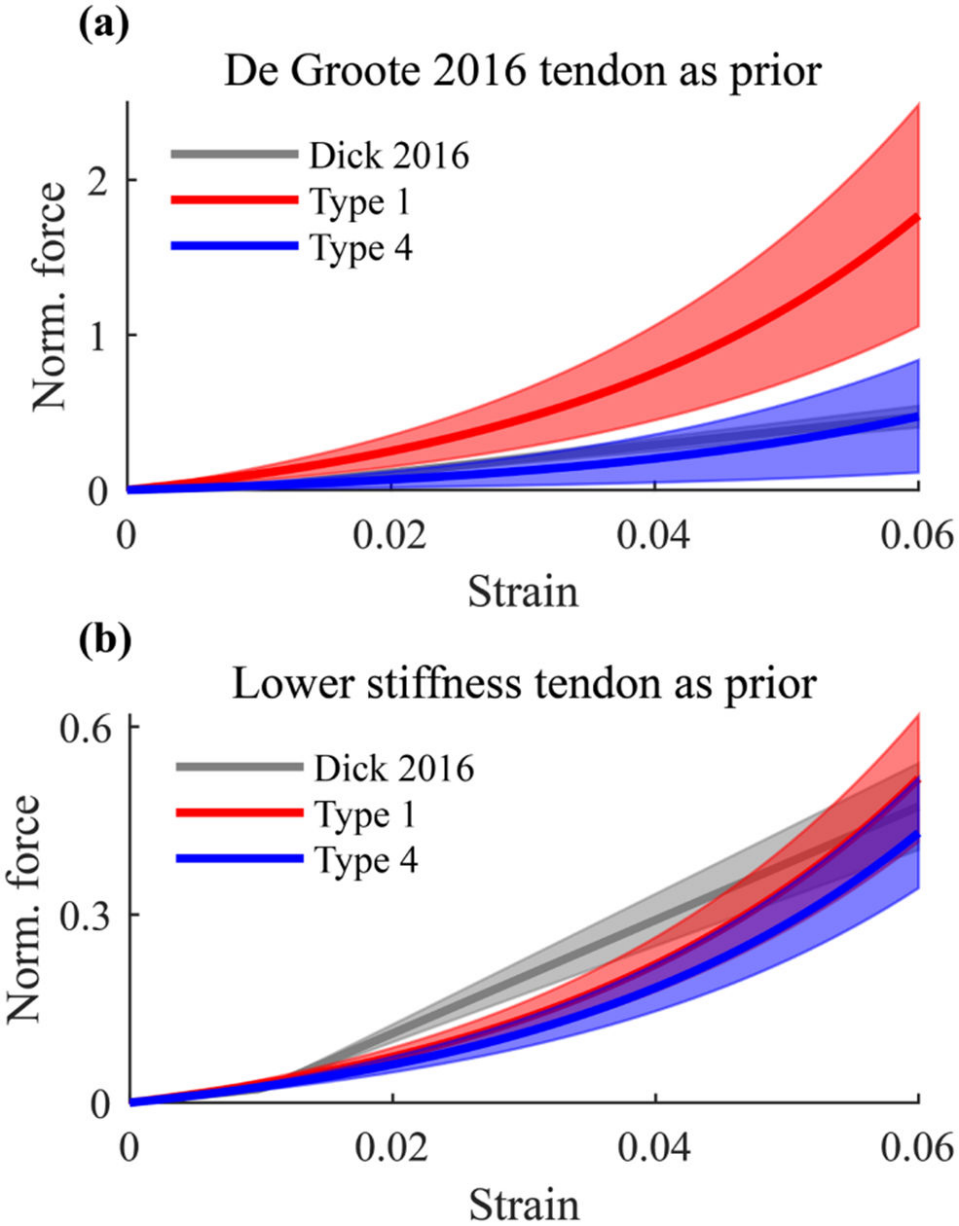
(a) Average tendon force-strain curve of Type 1 calibrated EMG-driven models (in red), and Type 4 calibrated EMG-driven models (in blue), using the De Groote et al. 2016 [24] tendon as prior (and $G_{t}\in$ (0.05, 1.5)), against experimental in vivo Achilles tendon force-strain curves from Dick et al. 2016 [31]. Results displayed as mean (solid lines) ± standard deviation (shaded area). (b) Average tendon force-strain curve of Type 1 calibrated EMG-driven models (in red), and Type 4 calibrated EMG-driven models (in blue), using the lower stiffness tendon as prior ($G_{t}=0.278\pm 30\%$), against experimental in vivo Achilles tendon force-strain curves from Dick et al. 2016 [31]. Results displayed as mean (solid lines) ± standard deviation (shaded area).

Using the De Groote 2016 tendon as prior, average tendon stiffness nRMSEs were always above 100% when reference tendon stiffness data were not used to calibrate the model, i.e., for Uncalibrated, Type 1, Type 2, and Type 3 models (Table II). Fig. 5(a) shows that the average tendon force-strain curve of the Type 1 model was stiffer than published experimental in vivo Achilles tendon force-strain curves [31]. When including reference tendon stiffness data to calibrate the model, i.e., Type 4 model, resulting tendon force-strain curves were more similar to reported in vivo data. Using a lower stiffness tendon as prior resulted in tendon force-strain curves that were similar to in vivo data, regardless of whether the EMG-driven model was calibrated without reference tendon stiffness data, e.g., Type 1, or with reference tendon stiffness data, i.e., Type 4 (Fig. 5(b)).

**Fig. 6.**
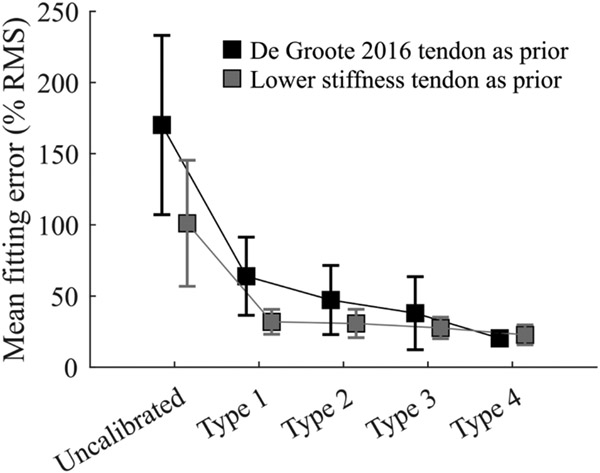
Mean fitting errors, i.e., root-mean-squared error normalized by reference root-mean-square expressed as a percentage, across all estimated biomechanical variables, i.e., ankle torque, ankle stiffness, muscle stiffness, tendon stiffness, and GM displacement, and across all subjects, for all five EMG-driven models defined per subject. Results of the EMG-driven models using the De Groote et al. 2016 [24] tendon as prior in black, and results using a lower stiffness tendon, i.e., $G_{t}=0.278$, as prior, in gray. Results displayed as mean (squares) ± standard deviation (vertical lines).

Fig. 6 shows the average nRMSE across all subjects and all five biomechanical variables for all defined EMG-driven models using both the De Groote 2016 tendon as prior and the lower stiffness tendon as prior. Average nRMSE decreased with increasing calibration complexity.

## IV. Discussion

We presented a model-based framework to estimate biological stiffness across anatomical levels that does not require ultrasonography nor external joint perturbations applied by a robotic manipulator. A new set of closed-form equations, in combination with muscle- and tendon-level reference data allowed, for the first time, the calibration of MTU characteristics such as the tendon force-strain and the muscle passive force-length relationships. A key result is that using a more compliant tendon, i.e., approximately four times more compliant than typically modeled, significantly improved stiffness estimation accuracy across anatomical levels in an EMG-driven model calibrated solely using reference joint torque.

First, we used a standard tendon force-strain curve (adapted from De Groote 2016) as a prior in our simulations. We demonstrated that a calibrated EMG-driven musculoskeletal model, i.e., Type 4 model, with a single set of MTU parameters can accurately estimate biomechanical variables across multiple anatomical levels (average nRMSE ≈ 20%, Fig. 4, Table II). Our results showed that with a standard calibration attempting to solely fit joint torques, i.e., Type 1 model, even though ankle torque was closely matched (nRMSE ≈ 5%), the underlying muscle- and tendon-level biomechanical variables were not agreeing with experimental measurements (Fig. 4). This represents a clear example of muscle redundancy, where a given joint torque can be obtained by infinite combinations of underlying muscle states. Furthermore, redundancy within a MTU could also be defined, as a certain MTU force can underlie infinite combinations of muscle and tendon stiffness. Our results could have broad implications in the context of established or emerging modeling frameworks such as OpenSim [7], [32], AnyBody [33], or MyoSuite [34], which currently disregard muscle, tendon, and joint stiffness.

We then compared the tendon force-strain curves of two different calibrated models, i.e., Type 1 and Type 4, to previously published in vivo data [31] (Fig. 5(a)) that, in line with other experimental studies [35], [36], reported rather compliant Achilles tendons, i.e., stiffness between 150–190 Nmm^−1^. Our results showed that calibrations that are not informed by reference tendon stiffness result in tendon force-strain curves that are stiffer than in vivo data, suggesting that the standard tendon force-strain relationships that are normally used in musculoskeletal modeling are too stiff. This finding is also supported by the fact that reference tendon stiffness could only be matched using a more compliant tendon force-strain relationship. The experimental measurements of tendon stiffness used here have previously been validated against in vivo measurements and direct measures from cadaveric samples [11]. It was demonstrated that outside of the lowest force levels (<100 N), estimated tendon stiffness was within one standard deviation of previous measures [35], [37]. In movements where tendon stiffness is greater than muscle stiffness, such as the movement investigated in this study, a stiffer tendon force-strain curve does not affect the estimation of joint-level biomechanical variables, such as joint torque and stiffness. Nevertheless, it leads to muscle- and tendon-level estimates that are not physiologically consistent. Conversely, in movements where tendons are more compliant than muscles, and thus dictate joint stiffness, such as standing [38], a stiffer tendon will lead to biased joint stiffness estimations.

Our novel modeling formulation enabled the calibration of $G_{t}$, i.e., a newly introduced model parameter that scales tendon stiffness. Consequently, when we used reference tendon stiffness profiles to inform the model calibrations, the resulting tendon force-strain curves were similar to in vivo data. In this way, not only joint-level biomechanical variables were closely matched, but also tendon- and muscle-level variables (Fig. 4, Table II). This example highlights the importance of combining data obtained from an experimental approach with a musculoskeletal modeling framework. We obtained a calibrated model that can accurately estimate biomechanical variables across multiple levels in a noninvasive way. Moreover, we gained access to biomechanical variables that were or could not be measured experimentally, such as the displacement of the SO or GL, or tendon strain.

We used the results of Fig. 5(a) to define a lower stiffness tendon force-strain curve with a narrower calibration range. In this way, regardless of whether or not the calibration was informed by reference tendon stiffness data, the resulting calibrated tendon force-strain curves were similar to in vivo data (Fig. 5(b)).

Regardless of what tendon force-strain curve was used as prior, overall fitting error decreased with increasing calibration complexity (Fig. 6), reaching an accuracy of approximately 20% with respect to the reference, which is in line with previously published studies [15], [39]. This suggests that an EMG-driven model based on the Hill-type muscle model with a single set of MTU parameters may be calibrated to fit forces and stiffness across anatomical levels. Moreover, our results also indicate that, the richer the data set that is used to inform the calibration, the more physiologically consistent the calibrated model is. However, since only one optimization per calibration type was performed, convergence to the optimal set of parameters cannot be assured, and future work should address this limitation by running multiple iterations of the same calibration. By improving accuracy across multiple biomechanical variables we increase our confidence in the model, and we are getting closer to having models that can be used in rehabilitation.

Moreover, using a more compliant tendon as prior significantly improved overall estimations even when the model was calibrated only using ankle torque (average nRMSE ≈ 32%, Table II, Fig. 6), in contrast to using the De Groote tendon as prior (average nRMSE ≈ 64%). Furthermore, using a more compliant tendon as prior yielded similar results for Type 1, Type 2, and Type 3 models, suggesting that using a more physiologically consistent tendon might lead to accurate models even when calibrated solely on joint torque, and informing the calibration with more biomechanical variables might not necessarily improve estimation capabilities. Therefore, translation to clinical use might be facilitated as less equipment will be needed to obtain reference data to calibrate the model.

This work entails some limitations. To match the assumptions made to obtain reference data, the proposed EMG-driven musculoskeletal model included three plantar flexor MTUs. Moreover, a constant moment arm of 51.3 mm was used for all subjects and MTUs, while MTU length was obtained using MTU-specific B-splines (Section II-D2). This modeling choice is valid as the computed B-splines were quasi-linear in the ankle angle range considered in this study. Future work should use subject- and angle-specific moment arms to compute stiffness following both the experimental and the EMG-driven modeling approaches.

In this study we estimated stiffness using a Hill-type muscle model in dynamic conditions. While the proposed model-based approach is generalizable to any joint and degree of freedom, in this article we focus on the ankle joint due to a lack of experimental stiffness profiles across anatomical levels of other joints and muscles. As soon as experimental data from other muscles and joints are available, future work should assess the extrapolation capabilities of our proposed method. Our results suggest that muscle and tendon stiffness, as well as GM displacement, could be estimated in dynamic conditions using a calibrated Hill-type muscle model that does not explicitly model short-range stiffness or history-dependent muscle properties. However, the calibrated models of some participants, e.g., subjects 3 and 4, displayed larger errors in muscle stiffness (Fig. 4), and further analyses are required to understand if the cause of the mismatch is the proposed modeling formulation or the experimental reference data. Whether a Hill-type muscle model with a single set of parameters can be used to estimate stiffness in both dynamic and static or postural conditions remains unknown, and future work should address this challenge. Estimating stiffness during static conditions might require explicit formulations of short-range stiffness [40] in parallel with the dynamic-range stiffness formulation used in this study, tuning the muscle active force-length curve as a way to modulate the muscle’s short-range stiffness, or the addition of history-dependent muscle properties [41].

Furthermore, this study did not investigate the sensitivity of the model calibration to the different MTU parameters, that were allowed to vary widely during calibration (Section II-E) to simultaneously fit multiple biomechanical variables. Despite the large ranges for accepted parameter values, the penalties that were defined in the calibration (Section II-D5) ensured that all muscles were operating at physiologically plausible lengths. Moreover, we found that the soleus’ optimal fiber length was always smaller than that of both gastrocnemii, which is in line with previous imaging studies of the calf muscles [42]. Future work should refine MTU parameter boundaries in such a way that optimal calibrations are achieved with a minimal reference data set.

Lastly, one assumption of the experimental data is that the estimates of muscle and tendon stiffness are estimates of the net stiffness of the triceps surae and Achilles tendon despite only making ultrasound measurements of the GM MTJ. This assumption has previously been validated, as we have found that the estimates during active contractions were similar when imaging the GM, GL, and SO MTJs [11]. While the proposed ankle musculoskeletal model comprises three separate MTUs (Section II-D) that share the total load, only the sum of their stiffnesses (Section II-E) is compared against the reference data in this study. Future work should investigate how individual muscles contribute to the net joint torque and stiffness in more complex situations where the knee and ankle are free to move.

## V. Conclusion

We present an EMG-driven modeling framework that can be calibrated and validated across multiple anatomical levels, i.e., joint, muscle, and tendon levels. Our results emphasize the importance of validating complex musculoskeletal models across different anatomical levels, as we demonstrate that the tendons that are normally used in musculoskeletal modeling are too stiff. We show that more compliant tendons are needed to better capture human biomechanics at the joint, tendon, and muscle levels. Calibrated musculoskeletal models informed by sensor-based measurements of biological signals give access to an augmented range of biomechanical variables across anatomical levels that might not be easily measured or estimated with sensors alone, i.e., by measuring joint-level biomechanical variables, a calibrated musculoskeletal model can provide insights on muscle and tendon dynamics. We envision that our innovative approach, which combines expertise from different scientific communities, will eventually bridge the gap between precise measurements from constrained experiments and computational models able to simulate functional conditions relevant to neurorehabilitation.

## Figures and Tables

**TABLE I:** Model Calibration Types Defined in This Study

EMG-driven model	Reference data used to calibrate the model			
	Ankle torque	Ankle stiffness	GM displacement	Tendon and muscle stiffness
Type 1	Yes	No	No	No
Type 2	Yes	Yes	No	No
Type 3	Yes	Yes	Yes	No
Type 4	Yes	Yes	Yes	Yes

**TABLE II:** Average Root-Mean-Square Error Normalized by Reference Root-Mean-Square Across All Subjects

EMG-driven model	Ankle torque	Ankle stiffness	GM displacement	Tendon stiffness	Muscle stiffness	Average error
Uncalibrated	232.0 (148.4)	49.7 (17.2)	61.6 (13.7)	439.9 (173.5)	67.5 (50.6)	170.1 (63.0)
	169.2 (121.8)	55.4 (22.9)	38.2 (12.5)	174.5 (88.9)	68.3 (33.0)	101.1 (44.3)
Type 1	5.2 (1.7)	19.3 (6.5)	38.9 (8.7)	197.5 (134.2)	58.7 (17.7)	63.9 (27.4)
	6.3 (3.6)	21.0 (8.7)	37.2 (11.6)	46.6 (23.0)	48.2 (28.7)	31.9 (8.7)
Type 2	10.3 (5.6)	7.6 (2.5)	49.5 (14.1)	123.5 (102.7)	45.2 (46.9)	47.2 (24.3)
	11.4 (6.2)	8.3 (3.1)	38.7 (11.2)	53.4 (19.8)	41.6 (33.3)	30.7 (9.9)
Type 3	11.4 (6.6)	9.7 (6.3)	19.8 (13.4)	104.6 (127.5)	44.2 (8.0)	37.9 (25.7)
	11.4 (6.1)	8.9 (2.8)	22.7 (12.4)	53.7 (20.0)	41.1 (12.9)	27.6 (7.6)
Type 4	17.6 (5.8)	17.8 (8.1)	25.5 (8.1)	16.4 (4.2)	24.1 (15.0)	20.3 (4.7)
	19.6 (7.9)	16.2 (7.7)	30.5 (13.4)	18.1 (4.2)	29.3 (18.2)	22.7 (7.0)

Results expressed as a percentage and reported as mean (standard deviation). Results of the model with De Groote 2016 tendon as prior in black font, and results of the model with a lower stiffness tendon as prior in gray font.

**TABLE III:** Coefficients for Generic and Normalized Muscle-Tendon Force and Stiffness Relationships

Tendon force-strain and stiffness-strain	$a_{1}$	0.200
	$a_{2}$	35
	$a_{3}$	0.005
	$a_{4}$	0.238
Muscle active force-length and active stiffness-length	$b_{11}$	0.813
	$b_{21}$	1.070
	$b_{31}$	0.266
	$b_{41}$	−0.025
	$b_{12}$	0.509
	$b_{22}$	0.701
	$b_{32}$	0.018
	$b_{42}$	0.162
	$b_{13}$	0.095
	$b_{23}$	1.004
	$b_{33}$	0.309
	$b_{43}$	−0.042
Muscle force-velocity	$c_{1}$	1.739
	$c_{2}$	0.030
	$c_{3}$	0.095
Muscle passive force-length and passive stiffness-length	$d_{1}$	0.014

## Appendix A Hill-Type Muscle-Tendon Characteristics

Table III contains the values of the coefficients used in Eqs. 5, 7, 8, and 9.
